# Supplementary material for: Transcriptomic and metabolomic characterization of antibacterial activity of Melastoma dodecandrum
Source: Front Plant Sci. 2023 Sep 13;14:1205725. doi: 10.3389/fpls.2023.1205725 (PMC10525717; doi:10.3389/fpls.2023.1205725)
Supplement: Supplementary Figure 1 — Time-kill assay of M.dodecandrum extract against PAO1.The time-kill activity of 4 and 25 mg/mL of methanol plant extract respectively from M. dodecandrum leaves or equivalent volumes of DMSO was assessed over 24 hours, with data points from each biological replicate plotted. [file Image_1.pdf]

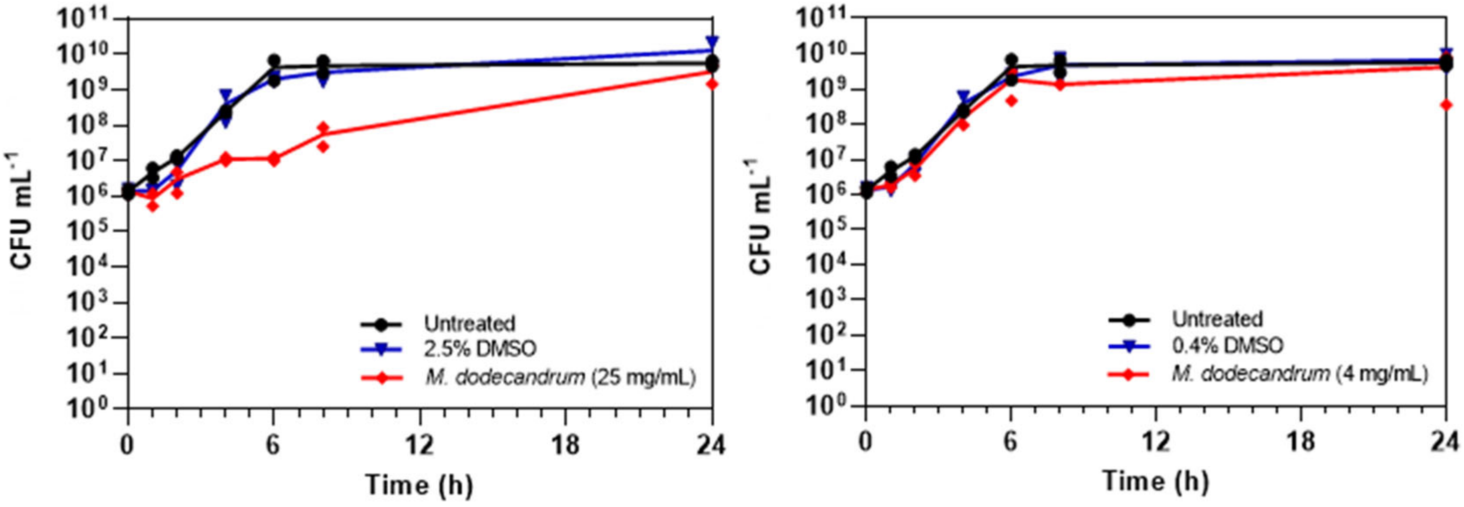

Figure S1 - Time-kill assay of M.dodecandrum extract against PAO1.The time-kill activity of 4 and 25 mg/mL of methanol plant extract respectively from M.

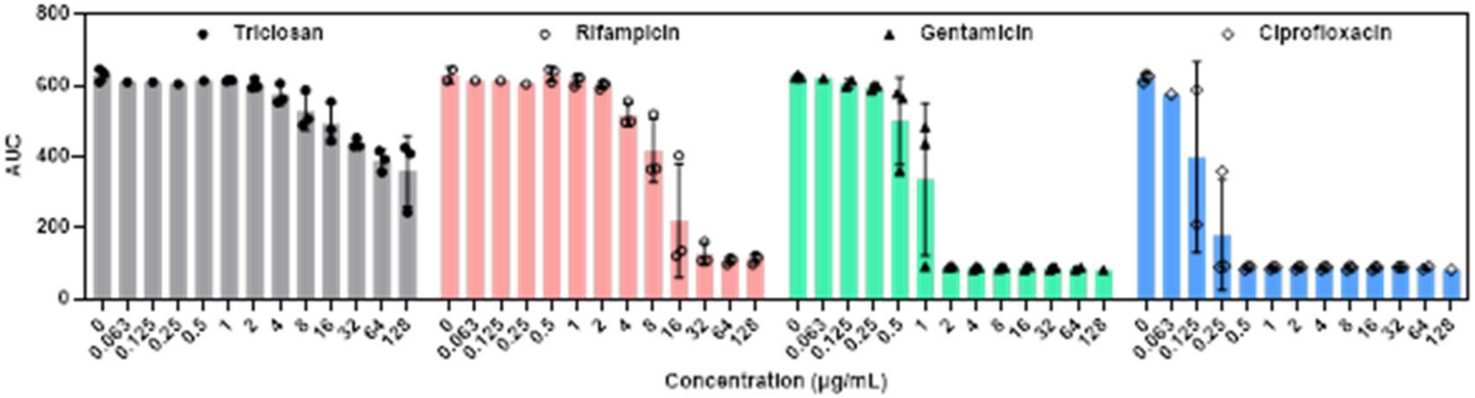

Figure S2 Effects of various antimicrobials on the growth of *P. aeruginosa* PAO1. Effects of the treatment of *P. aeruginosa* with 0 – 128 µg/mL of the antimicrobials triclosan, rifampicin, gentamicin, and ciprofloxacin were evaluated and the area under the curve was plotted. Each data point represents data from one independent biological replicate while error bars represent the standard error of the mean. At least two biological replicates were carried out.

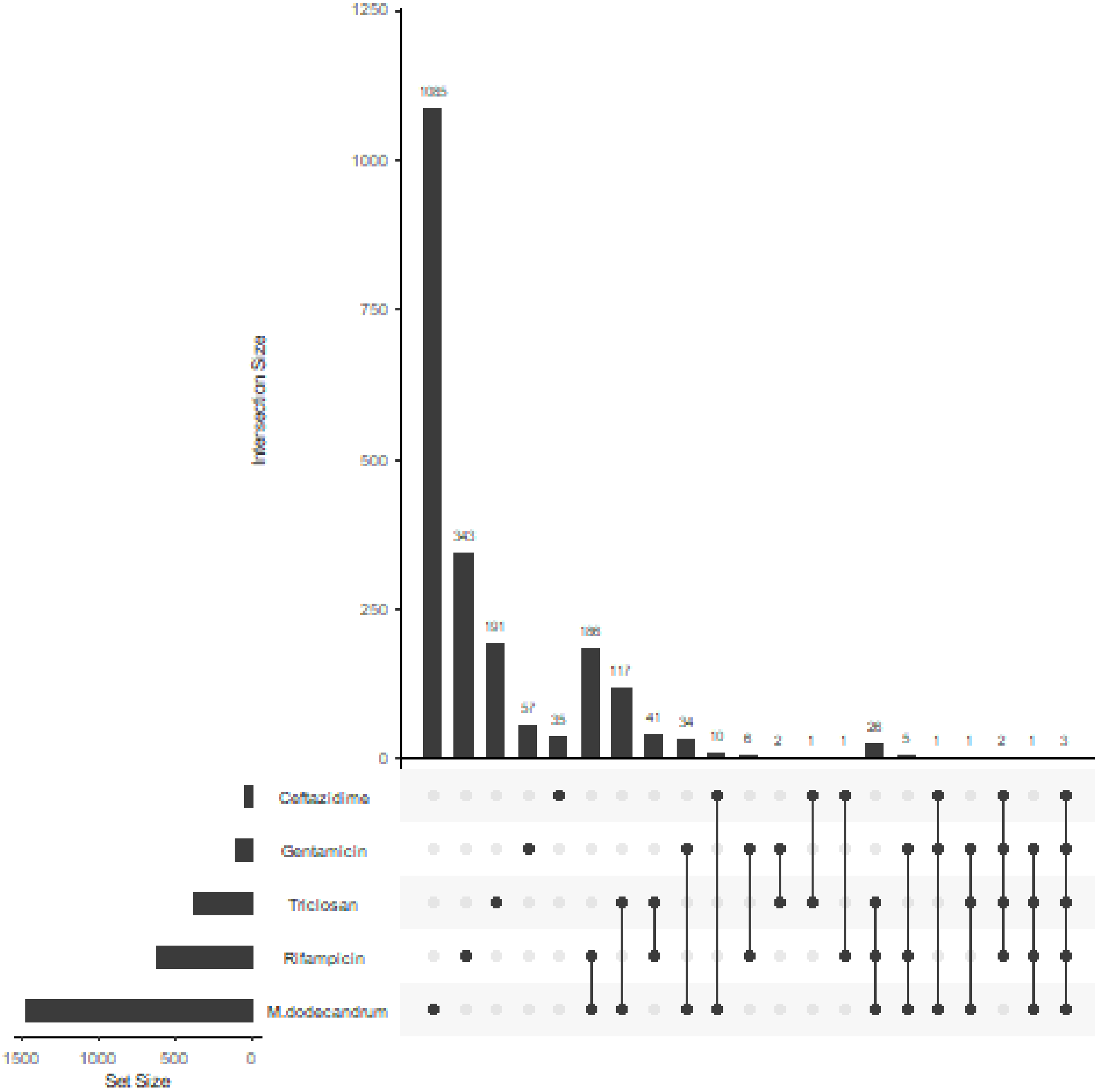

Figure S3 UpSet plot analysis of differentially expressed genes. The four antibiotics and *M. dodecandrum* plant extract is shown.

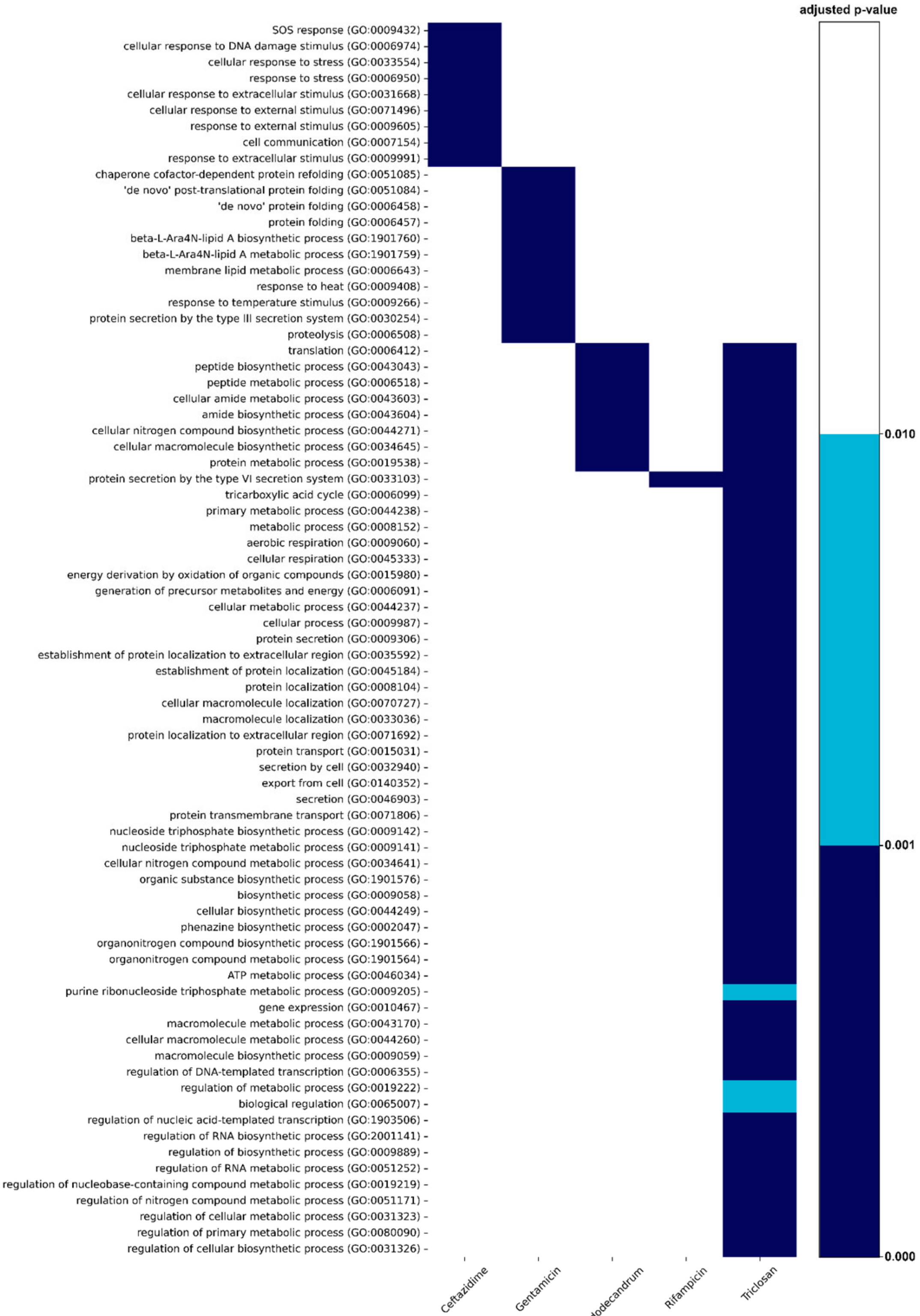

Figure S4 - Gene ontology enrichment analysis of the four antibiotics and M.dodecandrum extracts. Antibiotics and the extract are shown in columns, while significantly enriched gene ontology terms are shown in columns. Cell colors indicate the different significance levels of the enrichment.

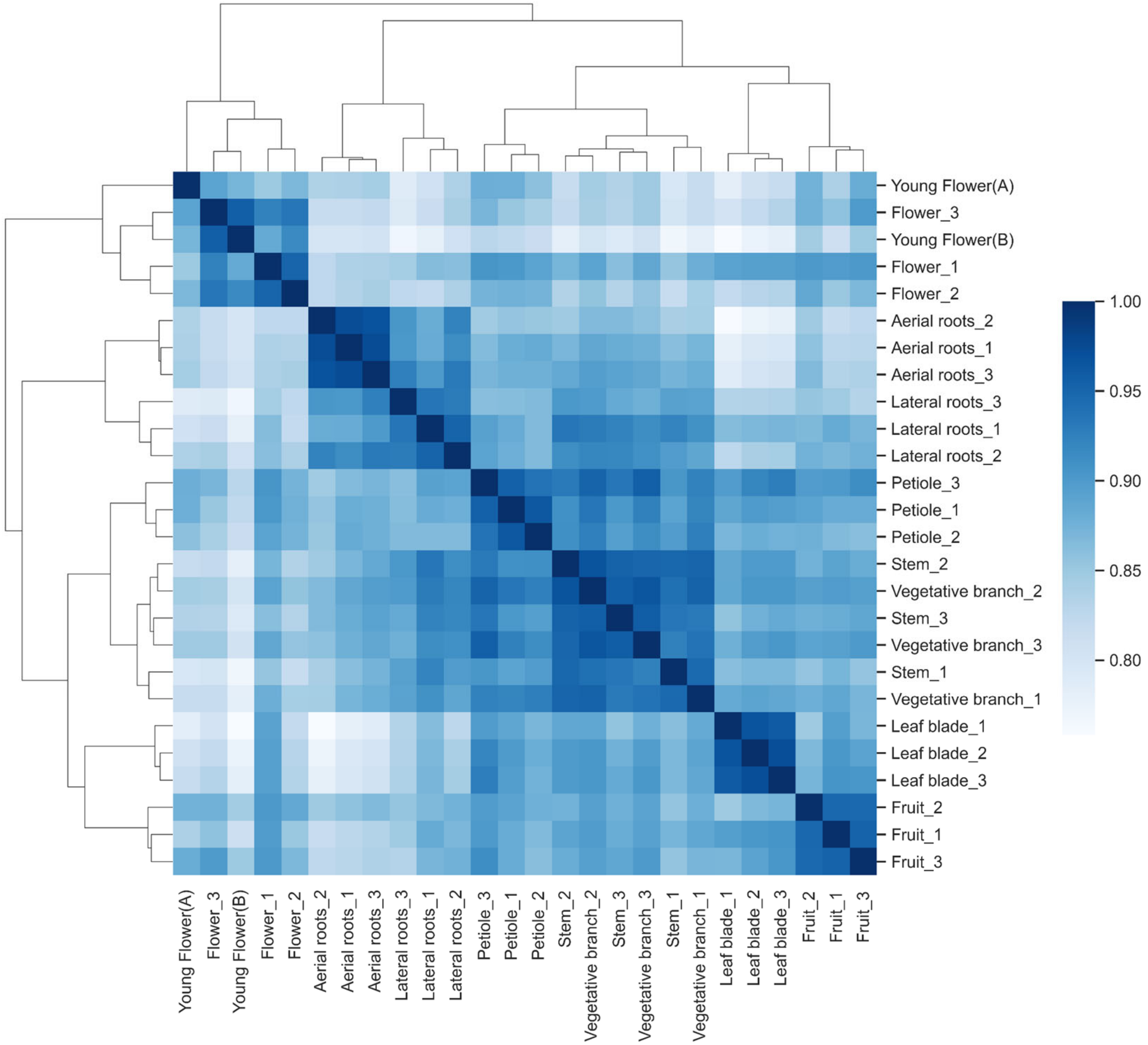

Figure S5. Hierarchical clustering analysis of gene expression data from Melastoma. Ward-linkage clustering based on Spearman correlation coefficient distance matrix.

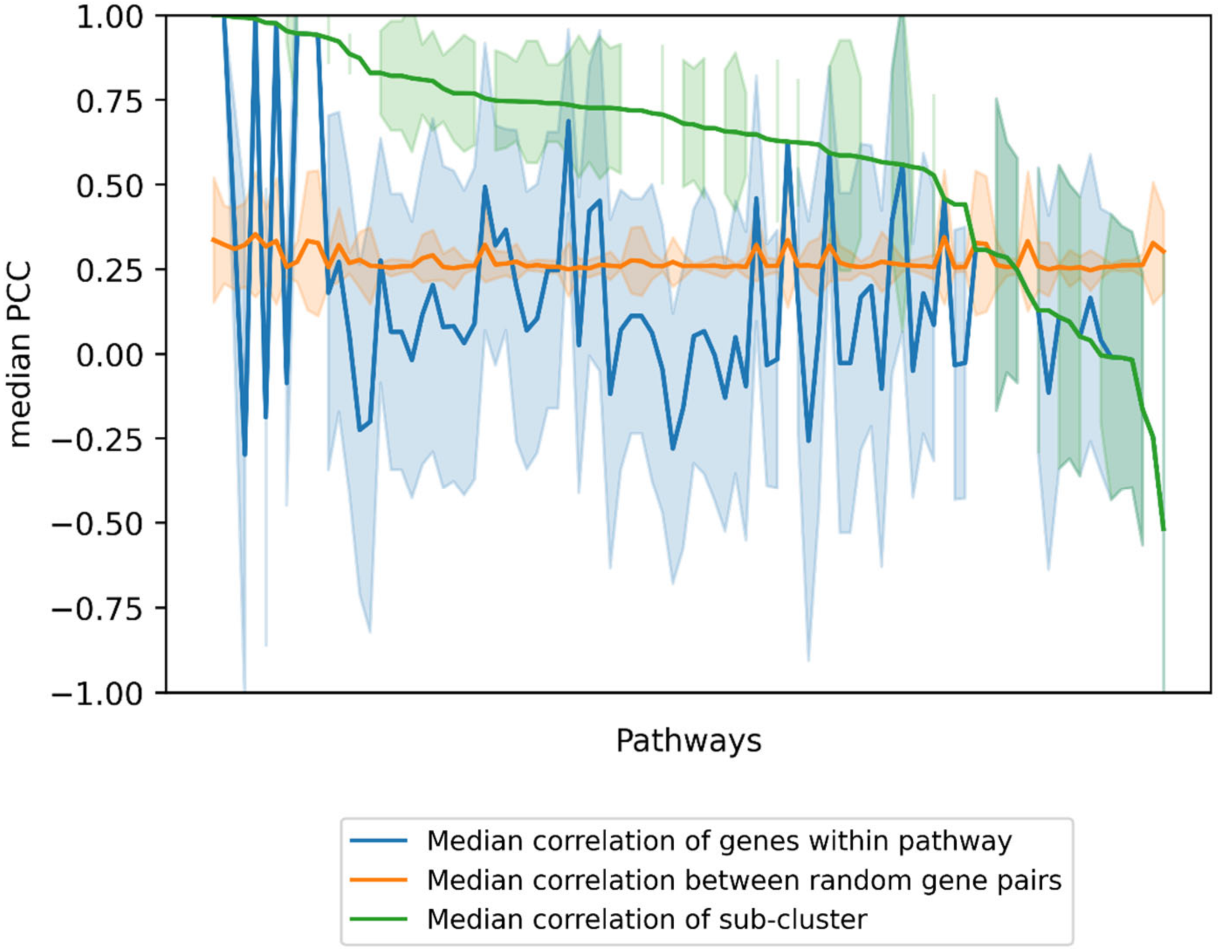

Figure S6. Correlation analysis of genes in the SM pathways of M.dodecandrum. Pathways without confidence interval bands comprise two genes only.
